# Wearable Inertial Sensors to Assess Gait during the 6-Minute Walk Test: A Systematic Review

**DOI:** 10.3390/s20092660

**Published:** 2020-05-06

**Authors:** Fabio Alexander Storm, Ambra Cesareo, Gianluigi Reni, Emilia Biffi

**Affiliations:** Scientific Institute, IRCCS “E. Medea”, Bioengineering Lab, 23842 Bosisio Parini, Lecco, Italy; eimber@hotmail.it (A.C.); gianluigi.reni@lanostrafamiglia.it (G.R.); emilia.biffi@lanostrafamiglia.it (E.B.)

**Keywords:** wearable sensors, MIMUs, 6-minute walk test, gait

## Abstract

Wearable sensors are becoming increasingly popular for complementing classical clinical assessments of gait deficits. The aim of this review is to examine the existing knowledge by systematically reviewing a large number of papers focusing on the use of wearable inertial sensors for the assessment of gait during the 6-minute walk test (6MWT), a widely recognized, simple, non-invasive, low-cost and reproducible exercise test. After a systematic search on PubMed and Scopus databases, two raters evaluated the quality of 28 full-text articles. Then, the available knowledge was summarized regarding study design, subjects enrolled (number of patients and pathological condition, if any, age, male/female ratio), sensor characteristics (type, number, sampling frequency, range) and body placement, 6MWT protocol and extracted parameters. Results were critically discussed to suggest future directions for the use of inertial sensor devices in the clinics.

## 1. Introduction

The 6-minute walk test (6MWT) is a simple, non-invasive, low-cost and reproducible exercise test used to evaluate endurance during self-paced, submaximal walk by measuring the distance walked within 6 minutes (6MWD) along a flat, straight course with a hard surface [1]. The 6MWT was originally developed for assessing exercise capacity in patients with cardiopulmonary diseases [2]. However, it has also been commonly used to measure functional status [3,4], as a predictor of morbidity and mortality [5,6], and for pre/post-treatment comparisons [7,8]. Besides the 6MWD, other useful outcome measures may be extracted from the 6MWT, including pulmonary function metrics [9], patient-reported fatigue at the beginning and end of the test using the Borg scale [10], and 6MWT work, calculated as the product of 6MWD and body weight [11]. However, a major limitation of the traditional 6MWT is that the only standardized outcome measure is the 6MWD, which does not take into account additional subject-specific factors such as gait length and width or body posture. Standard 6MWT performed in the clinics do not consider the granularity of overall gait patterns or body segment kinematics, and gait parameters are not commonly measured during the test. Moreover, it involves costs and some practical limitations such as the need for a dedicated space in the hospital and healthcare personnel to observe the test and to note down the measurements.

Accelerometers, gyroscopes and magnetometers are the most common wearable sensors used in human motion analysis and physical activity monitoring [12,13]. Accelerometers sense linear acceleration along one or several axis, and are the most widespread sensors used in ambulatory gait analysis, because they are miniaturized, low powered, durable, inexpensive, highly mobile, and readily available [14]. Modern gyroscopes measure angular velocity about one or several axes, with rotations around them commonly defined as Euler angles. Magnetometers sense amplitude and direction of the magnetic field exploiting the principle of the Lorentz force [15]. These sensors can be combined in devices called magneto-inertial measurement units, or MIMUs, which are gaining increasing popularity in human motion analysis. A recent review outlining the clinical impact of wearable sensors for gait analysis highlighted that MIMUs are the most widely used wearable technologies in the clinical field [16]. Readings obtained from the different sensors can be extracted and processed separately to obtain key features, or combined using fusion algorithms, such as Kalman filters, in order to obtain an estimated three-dimensional orientation [17].

Several MIMU-based methods have been proposed in the literature for the detection of gait events [18] and the computation of basic temporal parameters (stride and step duration, swing and stance phase duration). Spatial parameters (stride length, walking speed) can also be obtained by direct (i.e., integration of the accelerometer signal) or indirect (i.e., gait models) methods [19,20]. Gait dynamics, i.e., the study of stride-to-stride fluctuations in gait, includes basic variability metrics as well as advanced analytic methods, such as detrended fluctuation analysis [21], and can also be measured by means of MIMUs [22,23]. Wearable MIMU systems may also be able to estimate gait kinematics [24] and kinetics [25]. As a recent systematic review showed, new methods in artificial intelligence and machine learning are also increasingly being applied to wearable sensors data [26] because the latter exhibits many of the typical qualities of “big data” [27].

Despite the increasing body of evidence for the use of wearable sensing during gait, the use of MIMUs during standard clinical tests such as the 6MWT is not widespread. The well-defined setting of the 6MWT and its long duration compared to other walking tests represent favorable conditions to extract valuable information from both turn and straight walking, potentially increasing test efficiency. This study aims at reorganizing the existing knowledge by systematically reviewing a large number of papers focusing on the use of wearable inertial sensors for the assessment of advanced gait features during the 6MWT. It was not the interest of the authors to review papers focusing on energy expenditure, physical activity estimation or basic gait metrics extraction (i.e., step count). The objectives of this work are: (1) to select and perform a quality check on papers that assess gait during the 6MWT using wearable inertial sensors; (2) to identify the characteristics of the populations included in the studies; (3) to describe the features of the sensors used in terms of technical characteristics, number and location on the human body; (4) to indicate the main parameters extracted; (5) to suggest future directions for the use of inertial sensor devices in the clinics, especially fostering their testing and validation in the pediatric population.

## 2. Materials and Methods

For this review the authors followed the guidelines of the preferred reporting items for systematic reviews and meta-analyses (PRISMA) statement [28], and the original methodology of the systematic review was submitted to PROSPERO.

### 2.1. Search Strategy

Pubmed and Scopus search engines were accessed in November 2019 to identify articles measuring advanced quantitative parameters associated to gait with MIMU wearable sensors. The queries used for the database search within the title and/or abstract were the following: (“6MWT” OR “six-minute walk test” OR “six-minute walking test” OR “6MWD” OR "6-min walk test" OR "6-minute walk test") AND (“IMU” OR “inertial measurement unit” OR “MIMU” OR “magneto inertial measurement unit” OR “inertial sensor” OR “accelerometer” OR “wearable sensor” OR “smartphone” OR “activity tracker”). In addition, cross-referencing was applied to all included papers, in order to identify additional relevant studies. The literature search was conducted by F.S.

### 2.2. Study Selection and Quality Assessment

After completion of the preliminary electronic database search, one rater (F.S.) screened titles and abstracts and evaluated the suitability of each paper for inclusion in the present review. Papers were excluded if they: (i) Did not use a wearable inertial sensor during the 6MWT; (ii) were an abstract and/or were conference proceedings; (iii) were a review article or a case study; iv) were a study protocol; (v) were not written in English; (vi) were not ranked on Thomson Reuters; or (vii) were published before 2010. In addition, papers were excluded from further quality assessment if they were out of topic with respect to the aims of the present review i.e., the study of advanced gait parameters using wearable inertial sensors during the 6MWT. For example, studies were excluded if they focused on energy expenditure and physical activity intensity or used wearable sensors only to extract basic information such as the number of steps walked during the 6MWT.

Full-text publications that met the inclusion criteria were downloaded into Mendeley Desktop 1.19.4. Then, a quality assessment was performed for each article. The quality assessment checklist was based on similar published checklists used for systematical and/or meta-analysis reviews [29,30,31] and modified according to the specific needs and the topic of the present review. Items assessing internal (1, 3, 4, 6, 7, 9, 12, 13, and 14), external (2, 3, 5, 6, 8, 10, and 11) and statistical (15, 16, 17, and 18) validity were included [32,33] and are reported in Table 1. Each item of the checklist was given a score of 1, 0.5 or 0 corresponding to “met”, “partially met” or “not met”, respectively. The total score was then computed as the sum of scores of all the items in the checklist. Two authors assessed the quality of the included papers. The papers scoring above 10/15 corresponded to a “high quality” publication, those scoring between 9.5/15 and 5/15 were regarded as “medium quality” publications and those scoring below 5/15 were considered “poor quality” articles [34]. At the end of the quality assessment, Cohen’s kappa statistic [35] was used to compute the reliability of the agreement between the two raters, and the average of the scores was taken for the final quality assessment outcome. This statistic is used to measure inter-rater reliability for categorical items [36]: values ≤ 0 indicate no agreement, 0.01–0.20 none to slight, 0.21–0.40 fair, 0.41–0.60 moderate, 0.61–0.80 substantial, and 0.81–1.00 almost perfect agreement.

### 2.3. Data Extraction

The following information was collected from each paper: (i) aim, (ii) study design, (iii) subjects enrolled (number of patients and pathological condition, if any, age, male/female ratio), (iv) sensor characteristics (type, number, sampling frequency, range), (v) sensor body placement, (vi) length of the 6MWT walkway, (vii) calibration and filtering of raw data, (viii) parameters extracted, and (ix) synthetic conclusions.

## 3. Results

**Figure 1:**
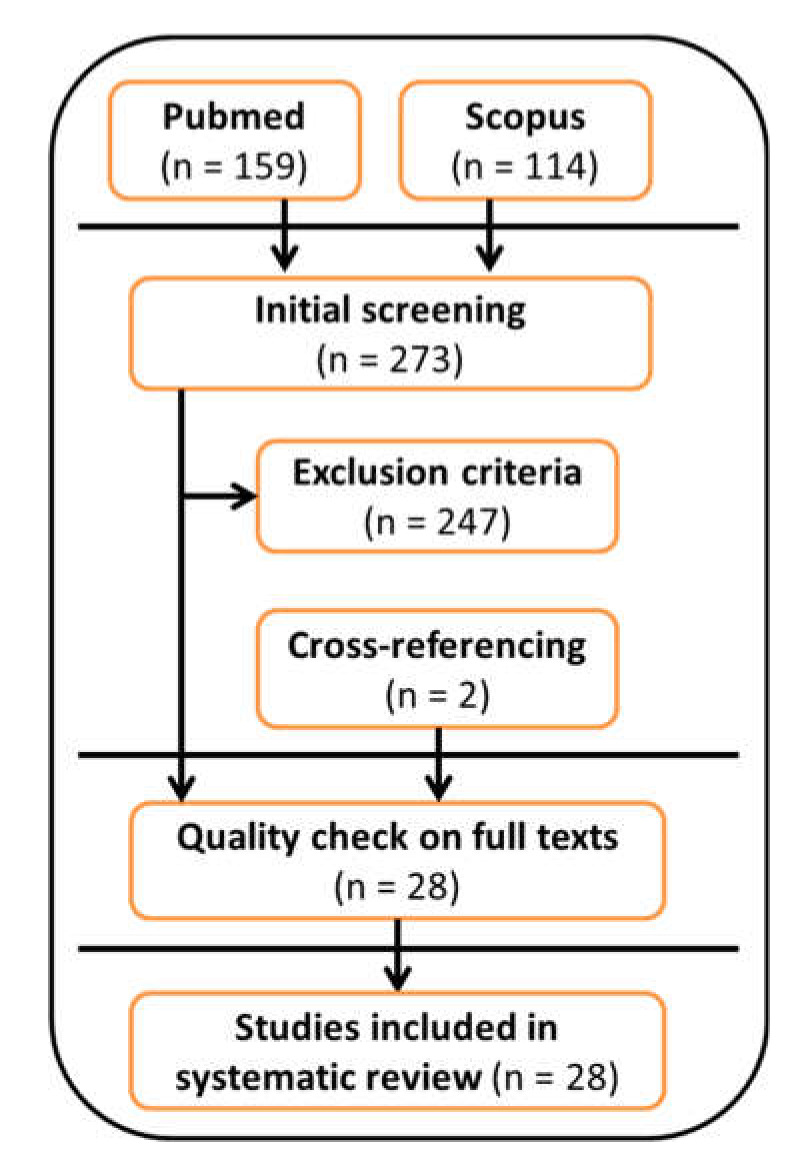
Flowchart of the systematic review process.

A flowchart of the systematic review process is reported in Figure 1. A total of 273 articles were included in the initial screening. A total of 232 articles were excluded after applying the exclusion criteria because they: (i) did not use a wearable inertial sensor during the 6MWT (168 papers); (ii) were an abstract and/or were conference proceedings (15 papers); (iii) were a review article or a case study (12 papers); (iv) were a study protocol (10 papers); (v) were not written in English (3 papers); (vi) were not ranked on Thomson Reuters (4 papers); or (vii) were published before 2010 (20 papers). In addition, 15 papers were excluded because they were out-of-topic, and two papers were included after cross-referencing. The remaining 28 articles were reviewed in full-text form and were all included in the systematic review after quality assessment.

### 3.1. Quality Assessment

Internal, external and statistical validity of the 28 included papers were evaluated by two authors (F.S and E.B.). The summary of the quality assessment process is shown in Table 2. Overall, 20 papers were classified as “high” quality (71.4%) and 8 papers were classified as “medium” quality (28.6%). Detailed results are provided in the Supplementary Materials (Table S1). The inter-rater agreement, computed by means of the Cohen’s kappa, was equal to 0.69, suggesting a substantial agreement between raters. A detailed summary of the papers included in this review is reported in Table 2.

### 3.2. Aims and Study Design

The aims of the studies reflected the predominant exploratory and preliminary nature of most articles. Based on the information reported in the reviewed articles, most of the papers were pilot (n = 12) or validation studies (n = 12). In addition, there were 2 feasibility studies and 2 cohort studies.

### 3.3. Population Characteristics

Populations with a variety of disease characteristics were investigated in the reviewed papers, the following being the most represented patient populations: people with multiple sclerosis (MS, 5 papers), chronic obstructive pulmonary disease (COPD, 5 papers), symptomatic lumbar spinal stenosis (sLSS, 2 papers) and cancer (2 papers). Healthy elderly and young healthy individuals were reported as studied populations in 2 papers each. Of the 28 papers, 12 (43%) included a healthy control group. Further details are reported in Table 3.

### 3.4. Sensor Characteristics

The authors of the papers included in the review used a variety of sensor types and supports to collect data, including research-grade MIMUs, triaxial accelerometers or gyroscopes, consumer-based sensors and smartphones with embedded sensors. All of the 28 included papers collected signals from at least one triaxial accelerometer. In general, 14 papers (50%) processed data collected through triaxial accelerometers only, 8 papers (28.6%) also collected signals from triaxial rate gyroscopes, and 6 papers (21.4%) used full MIMU data. Mostly, sensors were embedded in commercial research-grade sensors (19 papers, 67.9%). Some authors used sensors embedded in commercial smartphones or similar devices (7 papers, 25.0%) or used in-house manufactured devices (2 papers, 7.1%). The sampling frequencies (SF) at which the sensors collected data varied widely across papers. The most recurring SF was 60 Hz (6 papers, 21.4%), followed by 128 Hz and 100 Hz (3 papers each, 10.7%). SFs of 400 Hz and 50 Hz were reported by two papers each (7.1%). SFs of 1000 Hz, 200 Hz, 32 Hz, 30 Hz and 10 Hz were reported by one paper each (3.6%). Finally, 3 papers (10.7%) reported more than one SF, and 4 did not report any (14.3%). The majority of the papers did not report any sensor range (19 papers, 67.9%). The accelerometer sensor range was reported in 9/28 papers, the rate gyroscope range was reported in 5/14 papers, while the magnetometer range was reported in 1/6 studies.

### 3.5. Sensor Number and Locations

**Figure 2:**
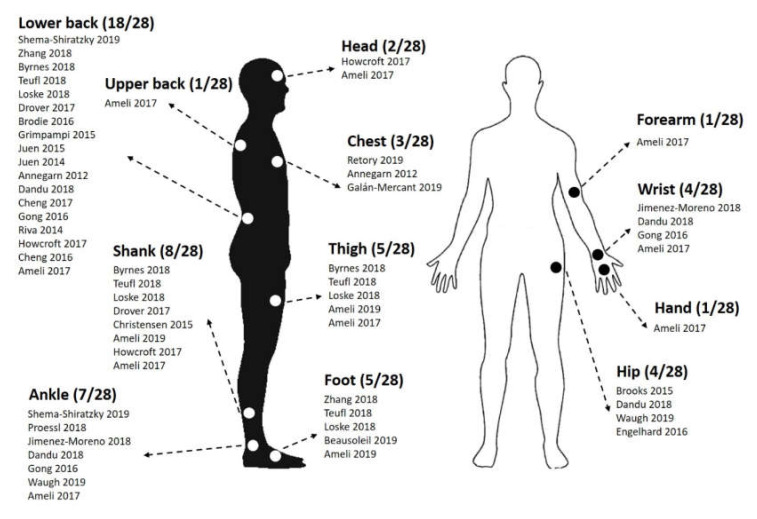
Sensor placement reported in the reviewed papers.

The relative majority of the papers (13/28, 46.4%) reported the use of a single sensor. Of the remaining 15 papers, 2 papers (7.1%) reported the use of 2 sensors, 4 papers (14.3%) reported 3 sensors, 2 papers (7.1%) reported 4 sensors, 1 paper (3.6%) reported 5 sensors, 2 papers (7.1%) reported 6 sensors, 3 papers reported 7 sensors (10.7%) and 1 paper (3.6%) reported the use of 17 sensors. Overall, the majority of the studies (64.3%, 18/28 papers) reported placing at least one wearable sensor on the lower back, with the preferred locations being at the level of the lower back (L3, L5 and the sacrum). Shanks and ankles were also frequent locations chosen by researchers, with 8 (28.6%) and 7 (25.0%) papers, respectively. Further details on the placement of the wearable sensors used in the included articles are summarized in Figure 2.

### 3.6. 6MWT Characteristics

All 6MWT were performed indoors along a flat straight corridor with 180° turns, apart for one study that used a rectangular set of corridors [39]. Most studies used a 30 m long path (11 papers, 39.3%), 8 articles did not report the length of the path (28.6%), 3 papers reported 15–16 m (14.3%), 3 papers (10.7%) reported 23 m (75 feet), 2 papers reported a variable number of path lengths, and one paper reported a length of 25 m.

### 3.7. Parameters Extracted during the 6MWT

The assessed papers reported a wide variety of outcome parameters obtained from MIMU data. Pre-processing of the signals involved a number of different techniques. Calibration procedures were reported by only 6 papers [41,45,49,56,58,59] and raw-data filtering was reported by 10 papers. The majority used lowpass Butterworth filters, one paper used a band-pass filter and one paper explicitly processed raw data without filtering (see Table 2 for details). Data processing was performed mostly using Matlab (10 papers, 35.7%, [38,41,44,45,46,51,58,59,62,63]). Eight papers did not report the software used (28.6%, [37,39,40,48,53,56,60,64]), 4 papers used custom software (14.3%, [47,49,50,57]). The remaining 6 papers (21.4%, [42,43,52,54,55,61]) reported the use of other software.

Spatio-temporal parameters were reported by the majority of the papers (16, 57.1%), acceleration descriptive statistics were reported by 10 papers (35.7%), 5 papers reported frequency and kinematics measures (17.9%), 3 papers reported stability indexes or other type of measures (10.7%), gait events were reported by only one study. A summary of the outcome parameters with a brief description are shown in Table 4.

## 4. Discussion

This work demonstrated that a significant number of papers (high quality = 20) have been published assessing gait using wearable inertial sensors during the 6MWT. We obtained substantial inter-rater agreement for the assessment of the quality of the papers analyzed (Cohen’s kappa = 0.69) [35]. When performing the quality assessment, the authors tuned their scores according to the available literature. For example, small sample sizes of the reviewed papers reflecting their piloting nature were not scored low a priori, but only if the conclusions of the papers were not justified or supported by the number of subjects recruited.

Technical advances in MIMU research are allowing this technology to become a valid alternative to classic laboratory-based and clinical assessments. While many methods and metrics still need to be validated, in the near future it will be possible to extend quantitative measures outside the classic clinical environment, even including free-living data to inform diagnosis and treatment of diseases [65,66].

In the authors’ opinion, the benefits of using MIMUs and wearable sensors in general during the 6MWT are evident: a thorough and comprehensive assessment of gait can be performed without adding further burden to the patient. They provide an alternative to traditional gait analysis and postural control assessments which require expensive equipment, are time consuming and can provide detailed information only for a very limited number of consecutive gait cycles [67]. Considering the hundreds of steps taken during the 6MWT, the information that can be extracted from this clinical test appears to be very valuable. Further information could be gathered from measures obtained during turning, which have shown to be more sensitive to detect impaired mobility than gait speed or clinical measures [68].

Of the reviewed articles, the majority of the pilot studies aimed at comparing gait features between a clinical population and a healthy control group. The preliminary outcomes gathered from these studies will serve to inform larger scale studies. Validation studies aimed generally at addressing accuracy, reliability and robustness of outcome measures against a clinical scale or a validated gold standard. The most represented clinical population were patients with MS, where wearable inertial sensors were used to inform on additional aspects of gait, especially walking-related fatigue. The reviewed papers showed that gait fatigability measured using both traditional and novel outcome parameters was significantly correlated with physical fatigue, gait disability, and fall history [37,56,62]. In patients with chronic obstructive pulmonary disease, most studies aimed at validating MIMU-based outcomes to predict pulmonary function and transitions in oxygen saturation [57,64]. Research on healthy elderly subjects mostly focused on the prospective fall-risk prediction and classification of fallers and non-fallers [44,63]. MIMUs were also useful in evaluating postoperative improvements in patients with symptomatic lumbar spinal stenosis and people recovering from total knee arthroplasty [43,48]. Healthy individuals were mostly included in fundamental research studies exploring newly proposed metrics or for validation studies. These results confirm the exploratory nature of most studies using MIMUs during the 6MWT, and the variety of outcomes and approaches that this technology allows. Some of the reviewed studies also used “big data” and machine learning approaches for disease classification [57] and fall risk prediction [63]. Our review confirms, as concluded by a recent paper [26], that machine learning in the field of gait analysis using inertial sensors is still in its preliminary steps. Although only a limited amount of studies used these techniques, we believe that this approach has a great potential to support the clinics that is still unexplored. Surprisingly, none of the reviewed studies included a pediatric cohort. Studies on healthy and pathologic young populations are therefore needed to evaluate the feasibility and to validate this approach also in the developmental age.

Not surprisingly, accelerometers were the most common source of raw data, due to their low power consumption and widespread use in comparison to rate gyroscopes and magnetometers [14]. Researchers used a variety of commercial sensors. Research-grade sensors were the most frequent choice in hospital settings, while sensors embedded in smartphones were favored when data were collected at home or the aim of the study was to validate these methodologies for future at-home use. Sampling frequencies varied between 10 Hz and 1000 Hz. This large spread may be partially justified by the variety of outcome measures proposed in the reviewed articles. For example, studies on physiological tremors and impacts may require sensing accelerations at up to 25–60 Hz [69,70]. However, it is worth noting that although accelerations at the foot that occur during initial contact can reach up to 60 Hz [71], 99% of the acceleration power during walking is concentrated below 15 Hz [72], suggesting that collecting data at very high sampling frequencies may be unnecessary. Inertial sensing measurement range is generally not critical for gait measurement applications, since some accelerometers may reach a range of up to 100 g and rate gyroscopes reach ranges of up to 2000°/s. However, saturation may occur when the full scale is set to ±2 g [73]. Most studies did not report this information in the methodology sections. As already highlighted by a previous systematic review focusing on balance assessment using wearable sensors [29], there was also a general lack of information related to sensor calibration in the papers reviewed in the present work, with only six reporting a calibration procedure, and only one acknowledging the lack of calibration as a potential limit of the work [39]. Most sensor axes were manually aligned to the anteroposterior, mediolateral and vertical axes without additional procedures for the minimization of sensor misalignment. A few static and dynamic calibration techniques to reduce the risk of cross-talk have been proposed in the literature [20,74]. In fact, one of the reviewed articles proposes a novel approach to improve the quality of accelerometer data during a 6MWT [45]. Information on pre-processing of raw data was also generally scarce, with only a minority of papers reporting the use of digital filters. Among them, there was a general uniformity in using low-pass Butterworth filters, with cut-off frequencies ranging between 2.5 and 40 Hz. The high variability of cut-off frequencies is justified by the variety of parameters extracted by the authors of the papers, and the locations of the sensors on the human body. It has been shown that low cut-off frequencies attenuate inertial sensor signals, while less restrictive filtering may provide more movement-related signals, but with the risk of higher noise [75]. Future studies should include more technical details regarding pre-processing of raw data used to calculate gait metrics, justifying their choices in light of existing literature. The research protocols of the reviewed articles included data collected from sensors positioned at 12 different body locations. Waist-placement was often preferred for single sensor configurations, because the sensor is positioned close to the center of mass of the human body, and hence thought to better represent human motions [76]. While the reviewed articles mostly focused on lower limbs (thighs, shanks, ankles and feet), there is also increasing awareness that upper body movements play a crucial role during locomotion and may be impaired for people with a variety of pathologies [77]. Future research should also focus on upper body variables, proposed as potential markers of disease progression [78], and on investigating the correlation between sensor location and outcome parameters.

Overall, the reviewed articles followed the technical guidelines for the 6MWT [2]. Regarding the 6MWT path length, a multicenter study found no significant effect of the length of straight courses ranging from 15 to 50 m, but patients walked further on continuous tracks [79]. While all papers reporting the path length were within this range, for a number of papers the length was not mentioned. For this reason, the authors believe that studies reporting 6MWT evaluation should carefully describe details about test administration.

The most frequently used outcome measures were those in the spatio-temporal domain, especially cadence. This is not surprising since a large amount of literature concerning the development of algorithms for step detection and step counting has been published in the last decades [80,81,82,83]. Other common spatio-temporal measures were variability and symmetry metrics, both being associated to gait impairment and balance disorders. Quantification of specific gait phases was also performed. The correct discrimination of gait phases can be important to distinguish between normal and pathologic gait, and for the evaluation of recovery after interventions or rehabilitation [18].

In the reviewed articles, the first quartile of Fourier transform and the ratio of even/odd harmonics were the most frequent outcome measures in the frequency domain. Both metrics have been linked to instability and higher fall risk [84,85]. The use of frequency domain measures obtained from accelerometer signals is increasing especially in the field of balance assessments. These metrics may provide better insights into balance also during gait, particularly for neurological disorders such as ataxia and Parkinson’s Disease [86].

Among acceleration descriptive statistics, root mean square (RMS) was one of the most reported outcome measures. RMS is a statistical measure of the magnitude of acceleration, it is simple to compute and of clear clinical meaning; however, its high correlation with walking speed needs to be taken into account when experiments are performed at self-selected pace. To overcome this limit, Sekine and colleagues recently proposed the RMS ratio, which represents the ratio between RMS in each direction and the RMS vector magnitude [87].

Non-linear analysis of inertial sensor data has also been proposed in recent years. Local dynamic stability of the gait pattern measured by means of accelerometers was assessed by some of the reviewed articles using short-term Lyapunov exponents [60,63], which quantify stride-to-stride local stability, taking also into account how the locomotor control system responds to perturbations [88]. The reviewed studies showed that these outcome measures could be used during the 6MWT in a clinical setting to obtain valuable information, especially during the turning phases of the test.

The most common kinematic indexes reported in the reviewed articles were angles computed at the lower limb joints. These metrics were generally compared against a gold standard, such as optical motion capture system [41,45]. Measurement of 3D joint angles using MIMUs is still a developing field and often lacks reliability and validity. However, a full kinematic analysis during the 6MWT using wearable MIMUs has already been attempted and proved to be technically feasible [24,89]; this kind of analysis would add significant value to the assessment.

Other measures have been proposed that do not fit into the previous categories, for example Dandu and colleagues propose dynamic time warping (DTW) and warp scores, outcome measures specifically designed and fitted to the 6MWT to quantify progressive changes to walking patterns [56]. Byrnes and colleagues describe outcome measures starting from the definition of an attractor, which represents the mean cycle of all strides and may reflect specific differences in pathologic gait [40].

### Clinical Implications

Gait has been acknowledged as a biomarker in many pathological conditions and the variety of gait parameters provided by inertial sensors can help clinicians to enhance assessments of interventions, disease progression and rehabilitation programs.

This review showed that wearable sensors are a technical solution that can be adopted in clinical settings to complement traditional assessments of gait deficits. The 6MWT is a well-suited test for this purpose because of its relatively long duration. Reporting additional insights contributes to inform clinical decisions and benefits the health care system by increasing test efficiency.

For example, parameters obtained using the methods reviewed in the present study were sensitive to disability in post-stroke patients [39], were suggested as outcome parameters for surgical procedures in lumbar spinal stenosis [40], and were useful in assessing the evolution of lower limb amputees during rehabilitation [52]. This technology also provided objective markers of gait fatigability in patients with multiple sclerosis [37,62]. Deterioration of gait features during the 6MWT using MIMUs appears to be a particularly promising direction of research for many neurological disorders where motor fatigue is an important symptom.

None of the reviewed papers included a pediatric population. This may be surprising because the 6MWT is a common functional outcome measure for chronic pediatric conditions [90]. However, it is likely that majority of the research involves adult populations and this is reflected proportionally in the papers included in the present review. Future studies may investigate how analytics from MIMUs during this standard test may improve our understanding of disability and disease progression in neurodegenerative disorders such as ataxia and muscular dystrophies. This technology also paves the way for remote monitoring in more ecologically valid environments.

## 5. Conclusions

After a systematic literature search and a quality assessment, we reviewed the current knowledge on the assessment of gait during the 6MWT using wearable MIMUs to extract advanced gait parameters, highlighting the main objectives of the studies, describing the preferred sensors and body location, and the outcome measures used in the studies. The results suggest that MIMUs could be successfully used to obtain additional information of clinical significance and increase the meaningfulness of the 6MWT evaluation. Future works should focus on extending the clinical populations studied, with particular attention to the pediatric population, on the validation of additional outcome measures and on extending the use of machine learning approaches.

## Figures and Tables

**Table 1:** Quality assessment checklist of internal, external and statistical validity (IV, EV and SV).

Item	Description	Outcome
	**Aim of the work**	
**1**	Description of a specific, clearly stated purpose (IV)	1, 0.5 or 0
**2**	The research question is scientifically relevant (EV)	1, 0.5 or 0
	**Inclusion criteria (selection bias)**	
**3**	Description of inclusion and/or exclusion criteria (IV/EV)	1, 0.5 or 0
	**Data collection and processing (performance bias)**	
**4**	Data collection is clearly described and reliable (IV/EV)	1, 0.5 or 0
**5**	Data processing is clearly described and reliable (IV/EV)	1, 0.5 or 0
**6**	Algorithms are clearly described and referenced (IV/EV)	1, 0.5 or 0
	**Data loss (attrition bias)**	
**7**	Drop-outs <20% (EV)	1, 0.5 or 0
	**Outcomes (detection bias)**	
**8**	Outcomes are topic relevant (EV)	1, 0.5 or 0
**9**	The work answers the scientific question stated in the aim (IV)	1, 0.5 or 0
	**Presentation of the results**	
**10**	Presentation of the results is sufficient to assess the adequacy of the analysis (IV)	1, 0.5 or 0
**11**	The main findings are clearly described (IV)	1, 0.5 or 0
	**Statistical approach**	
**12**	Appropriate statistical analysis techniques (SV)	1, 0.5 or 0
**13**	Clearly states the statistical test used (SV)	1, 0.5 or 0
**14**	Actual probability values reported for the main outcomes (SV)	1, 0.5 or 0
**15**	Sufficient number of subjects (SV)	1, 0.5 or 0

^1^ Internal validity, IV; ^2^ external validity, EV; ^3^ both internal and external validity IV-EV; ^4^ SV. Scores of 1, 0.5 and 0 correspond to items “met”, “partially met” or “not met”, respectively. Adapted from [29].

**Table 2:** Summary of the main characteristics of the articles included in the review.

ID	Paper	Aim	Study Design	Population Characteristics, Age (mean ± SD) and Male/Female Ratio	Population Characteristics	Sensor Type	Sensor Number	Sensor SF	Sensor Range	Raw signal Filter (Cut-Off Frequency)
1	Shema-Shiratzky 2019 [37]	To determine which gait features become worse during sustained walking in people with MS	Pilot	58 relapsing–remitting MS (49.0 ± 10.0, 17/41)	EDSS 2–6	3D ACC and 3D GYRO (OPAL, Apdm)	3	128 Hz	± 16 g; ± 2000 deg/s	/
2	Retory 2019 [38]	To determine gait parameters in subjects with high or low body mass index	Validation	10 controls (43.8 ± 12.8, 3/7) 13 non-overweight (42.2 ± 13.6, 4/9) 29 overweight (43.8 ± 12.8, 4/25)	BMI < 25kg/m^2^ BMI > 30kg/m^2^	3D ACC (Nox-T3, Polygraph)	1	10 Hz	± 2 g	LP 5th order Butter. (2.5 Hz)
3	Zhang 2018 [39]	To propose and evaluate a gait symmetry index	Feasibility	16 post-stroke (54, range 23–74, 9/7) 9 controls (35, range 25–48, 5/4)	SIS 190-288	3D ACC and 3D GYRO (MTw Awinda, XSens)	3	100 Hz	/	LP 2nd order Butter. (10 Hz)
4	Byrnes 2018 [40]	To determine characteristics of the attractor for acceleration gait data	Feasibility	19 sLSS (73.8 ± 5.3, 11/8) 24 controls (59.9 ± 10.5, 9/15)	ODI 27.9% ± 16.9%	3D IMU (RehaGait system, Hasomed GmbH)	7	400 Hz	± 16 g; ± 2000 deg/s; ± 1.3 Gs	LP 4th order Butter.
5	Teufl 2018 [41]	To evaluate the performance of an algorithm for the calculation of 3D joint angles	Validation	28 healthy (24.0 ± 2.7, 13/15)	/	3D ACC and 3D GYRO (MTw Awinda, XSens)	7	60 Hz	/	/
6	Proessl 2018 [42]	To investigate agreement between smart device and IMU-based gait parameters during prolonged walking	Validation	20 healthy (25.0 ± 3.7, 13/7)	/	3D ACC (Ipod Touch, Apple)	1	100 Hz	/	/
7	Loske 2018 [43]	To check if gait quality improves postoperatively	Cohort	20 sLSS 20 controls (60.5 ± 11.4)	ODI 30.7% ± 16.3%	3D IMU (RehaGait system, Hasomed GmbH)	7	400 Hz	/	/
8	Drover 2017 [44]	To validate a novel wearable sensor based faller classification method	Validation	76 older adults (74.15 ± 7.0)	/	3D ACC (X16-1C, Gulf Coast Data Concepts)	3	50 Hz	/	/
9	Brodie 2016 [45]	To validate an adaptive filter designed to improve the quality of accelerometer data	Validation	5 MS (68 ± 8, 0/5) 13 controls (32 ± 6, 4/9)	EDSS 4.3 ± 1.0	3D IMU (OPAL, Apdm)	1	128 Hz	± 6 g; ± 2000 deg/s	LP 4th order Butter.
10	Grimpampi 2015 [46]	To assess the reliability of gait variability assessment in healthy older individuals based on lower trunk accelerations	Validation	29 older adults (84 ± 5, 5/24)	/	3D ACC and 3D GYRO (Freesense, Sensorize)	1	/	/	/
11	Brooks 2015 [47]	To develop and validate a self-administered 6MWT mobile application.	Validation	103 CHF and pHTN	/	3D ACC (iPhone 4s, Apple)	1	/	/	/
12	Christiansen 2015 [48]	To examine movement symmetry changes over the first 26 weeks following unilateral TKA	Pilot	24 unilateral TKA (65.2 ± 9.2)19 controls (61.3 ± 9.2)	/	3D ACC (Delsys)	1	1000 Hz	± 10 g	LP 4th order Butter. (40 Hz)
13	Juen 2015 [49]	To evaluate six machine learning methods to obtain gait speed during natural walking	Pilot	28 pulmonary disease (range 50–89, 12/16) 10 controls (age range 18–69, 3/7)	/	3D ACC (S5 and Galaxy Ace, Samsung)	1	60 Hz	/	/
14	Juen 2014 [50]	To monitor health status using smartphones	Pilot	30 COPD (53 ± 11, 3/27)	GOLD 1–2	3D ACC (Galaxy Ace, Samsung)	1	60 Hz	/	/
15	Annegarn 2012 [51]	To determine walking patterns during the 6MWT of COPD patients and healthy elderly subjects	Cohort	79 COPD (64.3 ± 8.9, 47/32) 24 controls (63.7 ± 5.9, 15/9)	GOLD 1–2-3–4	3D ACC (Minimod, McRoberts)	1	100 Hz	± 2 g	LP 4th order Butter. (20 Hz)
16	Beausoleil 2019 [52]	To quantify the evolution of gait parameters along a 6MWT in LLA population	Pilot	15 LLA (59 ± 12, 10/5)	/	3D ACC and 3D GYRO (Physilog 4, GaitUp)	2	200 Hz	± 3 g; ± 600°/s	/
17	Galán-Mercant 2019 [53]	To predict physical activity and functional fitness using deep learning	Pilot	17 older adults (83.26 ± 6.56, 3/14)	/	3D ACC (iPhone 4, Apple)	1	32 Hz	/	LP 5th order Butter. (16 Hz)
18	Ameli 2019 [54]	To objectively assess the effects of chemotherapy-induced fatigue on gait characteristics	Pilot	4 breast cancer (50 ± 2.5)	/	3D IMU (MTx, Xsens)	6	60 Hz	/	/
19	Jimenez-Moreno 2018 [55]	To compare accelerometry data between a DM1 cohort and healthy controls	Validation	30 MD1 (48, range 25–72, 20/10) 14 controls (32, range 23–47, 6/8)	/	3D ACC (GENEActiv, Activinsights)	4	/	/	/
20	Dandu 2018 [56]	To explore the physiological and clinical meaning of four objective measures of walking impairment.	Pilot	115 MS	Mild (EDSS 0–2.5), moderate (3.0–4.0) and severe ( > 4.0)	3D ACC (GT3X, ActiGraph)3D ACC and 3D GYRO (in-house)	6	30 Hz, 128 Hz	/	BP (1–3 Hz)
21	Cheng 2017 [57]	To validate a model for the prediction of pulmonary function, based on motion sensor data from mobile phones	Validation	25 COPD (76, range 55–95, 15/10)	GOLD 1–2-3	3D ACC (Galaxy S5, Samsung and Optimus Zone2, LG)	2	/	/	/
22	Ameli 2017 [58]	To study the effects of fatigue induced by chemotherapy on PPS of cancer patients	Pilot	4 cancer patients	/	3D IMU (MTx, Xsens)	17	60 Hz	/	/
23	Gong 2016 [59]	To propose a causality analysis method that may aid disease diagnosis	Pilot	28 MS (40.5 ± 9.4, 7/21) 13 controls (39.3 ± 10.3, 6/7)	Mild (EDSS 0–2.5) and moderate (3.0–4.0)	3D ACC and 3D GYRO (in-house)	5	128 Hz	± 16 g; ± 2000°/s	/
24	Riva 2014 [60]	To evaluate the influence of directional changes and SF on gait variability and stability measures	Validation	51 healthy (23 ± 3)	/	3D ACC and 3D GYRO (FreeSense, Sensorize)	1	100 Hz - 200 Hz	/	Signal used unfiltered
25	Waugh 2019 [61]	To propose an individualized model of gait	Validation	92 older adults (86 ± 5, 33/53)	/	3D ACC (X6-2, X6-2mini, X8m-3, X16-2, Gulf Coast Data Concepts)	3	40 Hz - 50 Hz	± 2, 8, or 16 g	LP 4th order Butter. (10 Hz)
26	Engelhard 2016 [62]	To discover and validate objective evidence of gait alteration using dynamic time warping	Pilot	96 MS (46, range 19–61, 13/73) 29 controls (40, range 19–54, 9/20)	Mild (EDSS 0–2.5), moderate (3.0–4.5) and severe (5.0–6.5)	3D ACC (ActiGraph GT3X)	1	30 Hz	/	/
27	Howcroft 2017 [63]	To identify the optimal wearable sensor type, location, and combination for prospective fall-risk prediction	Pilot	76 older adults (75.2 ± 6.6, 31/44)	Fallers and non fallers	3D ACC (X16-1C, Gulf Coast Data Concepts)	4	50 Hz	/	LP 5th order Butter. (12.5 Hz)
28	Cheng 2016 [64]	To propose a gait model to predict saturation categories	Validation	20 COPD (66.3, range 43–81, 9/11)	GOLD 1–2	3D IMU (Droid 4 Mini, Motorola)	1	60 Hz	/	/

SF: sampling frequency; MS: multiple sclerosis; EDSS: expanded disability status scale; BMI: body mass index; SIS: stroke impact scale; sLSS: symptomatic lumbar spinal stenosis; ODI: Oswestry Disability Index; CHF: congestive heart failure; pHTN: pulmonary hypertension; TKA: total knee arthroplasty; COPD: chronic obstructive pulmonary disease; LLA: lower limb amputee; MD1: myotonic dystrophy type 1; GOLD: Global Initiative for Obstructive Lung Disease Criteria; ACC: accelerometer; GYRO: rate gyroscope; IMU: inertial measurement unit; LP: low-pass; BP: band-pass; butter: Butterworth filter.

**Table 3:** Summary of the population types reported in the included papers.

Population	N (% of Articles)	Total N of Patients	N with Controls	Total N of Controls
Multiple sclerosis (MS)	5 (17.9%)	302	3	55
Chronic obstructive pulmonary disease (COPD)	4 (14.3%)	126	1	24
Healthy elderly	4 (14.3%)	214	0	/
Healthy	3 (10.7%)	99	0	/
Symptomatic lumbar spinal stenosis (sLSS)	2 (7.1%)	39	2	44
Cancer	2 (7.1%)	8	0	/
Unilateral total knee arthoplasty (TKA)	1 (3.6%)	24	1	19
Myotonic dystrophy type 1 (DM1)	1 (3.6%)	30	1	14
Overweight	1 (3.6%)	29	1	23
Healthy elderly fallers	1 (3.6%)	28	1	47
Pulmonary disease	1 (3.6%)	28	1	10
Post-stroke	1 (3.6%)	16	1	9
Congestive heart failure (CHF) or pulmonary hypertension (pHTN)	1 (3.6%)	103	0	/
Lower limb amputees (LLA)	1 (3.6%)	15	0	/
TOTAL	28	1061	12	245

N: number of papers

**Table 4:** Summary and definition of the main outcome parameters of the reviewed papers.

Measure	Type	Definition	References
Number of steps	Event	Local maximum of the filtered acceleration signal	[38]
Number of U-turns	Event	Threshold at 95th percentile of vertical acceleration lower RMS curve	[38]
Distance	Spatio-temporal	Distance walked within 6MWT	[47,49,50]
Gait speed	Spatio-temporal	Distance traveled divided by time taken (m/s)	[37,52]
Cadence	Spatio-temporal	Steps taken divided by given time interval (steps/s)	[37,42,43,44,45,46,51,52,63]
Stance time	Spatio-temporal	Time between heel strike and toe-off	[52]
Stride time SD	Spatio-temporal	Stride time is defined as the time between two consecutive heel-strikes of the same foot	[46,60]
Stride time variability	Spatio-temporal	Stride time SD divided by mean stride time (%)	[37,60]
Swing time variability	Spatio-temporal	Swing time SD divided by mean swing time (%). Swing time is defined as the time interval between toe-off and the subsequent heel-strike of the same foot	[37]
Step length	Spatio-temporal	Number of steps between 2 consecutive U-turns divided by time taken	[38]
Stance ratio	Spatio-temporal	Percentage of the gait cycle during which the foot is in stance phase (%)	[39]
Load ratio	Spatio-temporal	Percentage of the stance corresponding to loading phase defined as the time between heel strike and toe strike (%)	[39]
Foot flat ratio	Spatio-temporal	Percentage of the stance corresponding to the foot-flat phase (%)	[39]
Push ratio	Spatio-temporal	Percentage of the stance corresponding to push phase defined as the time between heel off and toe off (%)	[39]
Symmetry of foot pitch angular velocity	Spatio-temporal	Pearson correlation coefficient (-)	[39]
Symmetry of foot pitch angular velocity	Spatio-temporal	Mean absolute difference between each left and right signal sample of cycle n divided by the mean range of the signals in the cycle (-)	[39]
Coefficient of stride cycle repetition	Spatio-temporal	Sum of positive autocorrelation coefficients of the three axes as a function of t (-)	[39]
Coefficient of step repetition	Spatio-temporal	Norm of autocorrelation coefficients as a function of t (-)	[39]
Gait asymmetry	Spatio-temporal	Percentage difference between left and right leg gait cycles (%)	[43]
Width and length of Poincaré plots	Spatio-temporal	Width and length of the long and short axis of the stride duration elliptical data plots between successive gait cycles (-)	[60]
Flat foot ratio	Spatio-temporal	Foot flat time as a percentage of the whole gait cycle (s)	[52]
Minimal toe clearance	Spatio-temporal	Minimal toe clearance during the swing phase (m)	[52]
Stride regularity	Frequency	Unbiased and normalized autocorrelation coefficient at the second dominant period (-)	[37]
Step regularity	Frequency	Unbiased and normalized autocorrelation coefficient at the first dominant period (-)	[37]
First quartile of Fourier transform (FQFFT)	Frequency	Percentage of acceleration frequencies within the first quartile of an FFT frequency plot (%)	[44,63]
Ratio of even/odd harmonics (REOH)	Frequency	Ratio of acceleration signal in phase with stride frequency (-)	[44,60,63]
Peak frequency	Frequency	Frequency of greatest magnitude in spectrum (Hz)	[57,64]
Shannon enthropy	Frequency	Expected value of signal information	[57,64]
Root mean square (RMS)	Acceleration descriptive statistics	RMS of the accelerations in anteroposterior, mediolateral and vertical directions	[46,57,64]
Acceleration maximum	Acceleration descriptive statistics	Acceleration maximum of positive and negative axis direction	[44,63]
Acceleration mean	Acceleration descriptive statistics	Acceleration mean of positive and negative axis direction	[44,57,63]
Acceleration standard deviation	Acceleration descriptive statistics	Acceleration standard deviation of positive and negative axis direction	[44,57,63]
Acceleration coefficient of variance	Acceleration descriptive statistics	Acceleration mean divided by standard deviation	[57,64]
Interstride trunk variability	Acceleration descriptive statistics	Mean values of the unbiased autocorrelation coefficients of the three acceleration components	[46,51]
Initial peak acceleration	Acceleration descriptive statistics	Peak tibial acceleration after foot contact	[48]
Absolute symmetry index	Acceleration descriptive statistics	Absolute differences in initial peak acceleration between limbs (%)	[48]
Walking intensity	Acceleration descriptive statistics	Integral of the modulus accelerometer output	[51]
Resultant acceleration	Acceleration descriptive statistics	Square root of the sum of squared acceleration signals in AP, ML an V directions	[53]
Euclidean norm minus one	Acceleration descriptive statistics	Resultant acceleration minus one	[55]
Short-term lyapunov exponents	Non-linear indexes	Quantifies stride-to-stride local dynamic stability of walking	[60,63]
Recurrence quantification analysis	Non-linear indexes	Provides a characterization of a variety of features of a given time series, including a quantification of deterministic structure and non-stationarity, based on the construction of recurrence plots.	[60]
Multiscale entropy	Non-linear indexes	Quantifies the complexity or irregularity of a time series	[60]
Index of harmonicity	Non-linear indexes	Quantifies the contribution of the stride frequency to the signal power relative to higher harmonics	[60]
Sample entropy	Non-linear indexes	Negative logarithm of the probability that if two sets of simultaneous data points of length m have distance <r then two sets of simultaneous data points of length m + 1 also have distance <r	[37]
Path length	Kinematics	Ratio between the length of the real path of the foot in 3D space (including both stride length and width) and stride length of one cycle (% stride length)	[39]
Strike angle	Kinematics	Angle between the foot and the ground at heel strike in sagittal plane (deg)	[39]
Lift off angle	Kinematics	Angle between the foot and the ground at toe off in sagittal plane (deg)	[39]
Max angular velocity	Kinematics	Maximum pitch foot angular velocity during swing phase (deg/s)	[39]
Hip, knee, ankle joint, and pelvis angles	Kinematics	Joint angles using Euler angle decomposition (deg)	[41,45,54,58]
Causality index	Other	Sum of the number of significant relationships remaining after thresholding the pairwise causality matrix	[56]
Kernel density estimation (KDE) peak	Other	Peak of the density functions at 100 equally spaced inertial gait data amplitude values	[56]
Dynamic time warping (DTW) score	Other	Summarizes the degree of similarity between sequences following alignment	[56]
Warp score	Other	Summarizes the number of “warps”, or repetitions of samples, needed to achieve an optimal alignment between sequences.	[56,62]
Change in acceleration pattern between two conditions (δM)	Other	Difference between two attractors	[40]
Change in variability around the attractor (δD)	Other	Change in acceleration variability between conditions	[40]
Attractor-based index	Other	Product of δM and δD	[40]

## Supplementary Materials

The following are available online at https://www.mdpi.com/1424-8220/20/9/2660/s1, Table S1: Detailed results of the quality assessment conducted by each rater on the articles included in the systematic review.
