# Supplementary material for: Wearable Inertial Sensors to Assess Gait during the 6-Minute Walk Test: A Systematic Review
Source: Sensors (Basel). 2020 May 6;20(9):2660. doi: 10.3390/s20092660 (PMC7249076; doi:10.3390/s20092660)
Supplement: Supplementary file 1 [file sensors-20-02660-s001.pdf]

sensors

Supplementary

Wearable Inertial Sensors to Assess Gait during the 6-Minute Walk Test: A Systematic Review

sensors

Table S1. Detailed results of the quality assessment conducted by each rater on the articles included in the systematic review.

ID	Paper	Item 1		Item 2		Item 3		Item 4		Item 5		Item 6		Item 7		Item 8		Item 9		Item 10		Item 11		Item 12		Item 13		Item 14		Item 15	
		R1	R2	R1	R2	R1	R2	R1	R2	R1	R2	R1	R2	R1	R2	R1	R2	R1	R2	R1	R2	R1	R2	R1	R2	R1	R2	R1	R2	R1	R2
1	Shema-Shiratzky 2019	1.0	1.0	1.0	1.0	0.5	0.5	1.0	1.0	1.0	1.0	0.5	1.0	0.5	1.0	1.0	1.0	1.0	1.0	1.0	1.0	1.0	1.0	1.0	1.0	1.0	1.0	1.0	1.0	1.0	1.0
2	Retory 2019	0.5	1.0	0.5	1.0	0.5	0.5	1.0	1.0	0.5	1.0	0.5	1.0	1.0	1.0	0.5	1.0	1.0	1.0	1.0	1.0	0.5	1.0	1.0	1.0	1.0	1.0	0.0	0.5	0.5	1.0
3	Zhang 2018	1.0	1.0	0.5	1.0	0.0	0.0	1.0	1.0	0.5	1.0	1.0	1.0	1.0	1.0	1.0	1.0	1.0	1.0	1.0	1.0	1.0	1.0	1.0	0.5	1.0	1.0	1.0	1.0	1.0	0.5
4	Byrnes 2018	1.0	1.0	1.0	0.5	1.0	1.0	0.0	1.0	0.5	1.0	0.5	1.0	1.0	1.0	1.0	1.0	1.0	1.0	0.0	1.0	1.0	1.0	1.0	0.5	0.5	1.0	1.0	1.0	1.0	0.5
5	Teufl 2018	1.0	1.0	1.0	1.0	0.5	0.0	1.0	0.5	1.0	0.5	1.0	1.0	1.0	1.0	1.0	1.0	1.0	1.0	1.0	1.0	1.0	1.0	1.0	1.0	1.0	1.0	1.0	1.0	1.0	1.0
6	Proessl 2018	1.0	1.0	0.5	0.5	0.5	1.0	0.5	1.0	0.5	0.5	0.0	0.5	1.0	1.0	0.5	1.0	0.5	1.0	0.5	0.5	1.0	1.0	0.5	1.0	1.0	1.0	1.0	0.5	1.0	1.0
7	Loske 2018	1.0	1.0	1.0	1.0	1.0	1.0	1.0	1.0	0.5	0.5	0.5	0.5	0.0	0.5	1.0	1.0	1.0	1.0	1.0	1.0	1.0	0.5	1.0	1.0	1.0	1.0	1.0	1.0	0.5	1.0
8	Drover 2017	1.0	0.5	1.0	1.0	0.5	1.0	1.0	1.0	1.0	0.5	0.5	0.5	1.0	1.0	1.0	1.0	1.0	0.5	1.0	0.5	1.0	0.5	1.0	0.5	1.0	1.0	1.0	0.5	0.5	1.0
9	Brodie 2016	0.5	0.5	1.0	0.5	0.5	0.0	1.0	0.5	1.0	1.0	1.0	1.0	1.0	1.0	1.0	0.5	1.0	0.5	1.0	0.5	1.0	1.0	0.5	0.0	1.0	1.0	1.0	1.0	0.0	0.5
10	Grimpampi 2015	1.0	1.0	1.0	1.0	0.5	1.0	1.0	1.0	0.5	1.0	0.5	1.0	1.0	1.0	1.0	1.0	1.0	1.0	1.0	1.0	1.0	0.5	1.0	1.0	1.0	1.0	1.0	1.0	0.5	1.0
11	Brooks 2015	0.5	0.0	0.5	0.5	0.5	0.5	0.5	0.5	0.5	0.5	0.0	0.0	0.0	0.5	1.0	1.0	1.0	0.5	1.0	0.5	1.0	1.0	1.0	1.0	1.0	1.0	1.0	1.0	0.0	1.0
12	Christiansen 2015	0.5	1.0	0.5	0.5	1.0	1.0	0.5	1.0	0.5	0.5	0.5	0.5	1.0	0.5	1.0	1.0	1.0	1.0	0.5	0.5	0.5	1.0	1.0	0.5	1.0	1.0	1.0	0.5	1.0	1.0
13	Juen 2015	0.5	0.5	0.5	1.0	0.5	0.0	0.5	0.5	0.5	0.5	0.5	0.0	0.5	0.5	1.0	0.5	1.0	0.5	0.5	0.5	1.0	0.0	0.5	0.5	0.5	0.0	0.0	0.0	0.5	0.5
14	Juen 2014	0.5	0.5	1.0	0.5	0.0	0.0	0.5	0.5	0.5	0.0	0.0	0.0	0.5	1.0	1.0	0.5	0.5	0.5	0.5	0.5	0.5	0.5	0.5	0.5	0.0	0.5	0.5	0.0	1.0	0.5
15	Annegarn 2012	1.0	1.0	1.0	1.0	0.5	0.5	1.0	1.0	1.0	1.0	0.5	0.5	1.0	1.0	1.0	1.0	1.0	1.0	1.0	0.5	0.5	1.0	1.0	0.5	1.0	0.5	1.0	0.5	1.0	1.0
16	Beausoleil 2019	1.0	1.0	1.0	1.0	1.0	1.0	0.5	1.0	0.5	0.0	0.0	0.5	1.0	1.0	1.0	1.0	1.0	1.0	0.5	1.0	1.0	1.0	0.5	0.5	0.5	0.5	1.0	1.0	0.5	0.5
17	Galán-Mercant 2019	1.0	0.5	1.0	0.5	0.5	1.0	1.0	1.0	1.0	0.5	1.0	0.5	1.0	1.0	1.0	0.5	1.0	0.5	1.0	0.5	1.0	0.5	1.0	0.5	1.0	1.0	0.5	0.0	0.5	0.5
18	Ameli 2019	0.5	0.0	1.0	0.5	1.0	1.0	0.5	0.5	0.5	0.5	1.0	1.0	1.0	1.0	0.5	0.5	0.5	0.0	0.0	0.0	0.5	1.0	0.5	0.0	1.0	0.0	0.5	0.0	0.0	0.0
19	Jimenez-Moreno 2018	0.5	1.0	0.5	1.0	0.0	0.0	0.5	1.0	1.0	1.0	0.5	0.5	1.0	1.0	0.5	1.0	0.5	1.0	0.5	1.0	1.0	1.0	1.0	1.0	1.0	1.0	1.0	1.0	0.5	1.0
20	Dandu 2018	1.0	1.0	1.0	0.5	1.0	1.0	1.0	1.0	1.0	1.0	1.0	1.0	0.0	0.0	1.0	1.0	0.5	0.0	0.5	0.5	1.0	0.5	1.0	1.0	1.0	1.0	1.0	0.0	1.0	1.0
21	Cheng 2017	0.5	0.0	1.0	0.5	0.0	0.5	0.5	0.5	0.0	0.5	0.5	0.5	0.0	0.5	0.5	0.5	1.0	0.0	0.5	0.0	0.0	0.5	1.0	0.0	1.0	0.0	0.5	0.0	0.5	0.5
22	Ameli 2017	0.5	0.5	1.0	1.0	0.0	0.0	0.5	0.5	0.5	0.5	1.0	0.5	1.0	1.0	0.5	0.5	0.5	0.5	0.0	0.5	0.5	0.5	0.5	0.5	0.5	0.5	0.5	0.0	0.0	0.5
23	Gong 2016	0.5	1.0	1.0	0.5	0.5	0.0	0.5	0.5	1.0	1.0	1.0	1.0	1.0	1.0	1.0	1.0	1.0	0.5	0.5	0.5	0.5	1.0	0.5	1.0	1.0	1.0	0.0	0.0	0.5	0.5
24	Riva 2014	1.0	1.0	0.5	1.0	0.5	0.5	1.0	1.0	1.0	1.0	1.0	1.0	1.0	1.0	1.0	1.0	1.0	1.0	1.0	0.5	1.0	1.0	1.0	1.0	1.0	1.0	1.0	1.0	0.5	1.0
25	Waugh 2019	1.0	0.5	0.5	1.0	0.0	0.0	0.5	0.5	0.5	1.0	0.5	1.0	1.0	1.0	1.0	0.5	1.0	0.5	0.5	0.5	0.5	0.5	0.5	0.0	0.5	0.0	1.0	0.0	0.5	1.0
26	Engelhard 2016	1.0	1.0	1.0	1.0	0.0	0.5	1.0	1.0	1.0	1.0	1.0	1.0	1.0	1.0	1.0	1.0	1.0	1.0	0.5	1.0	0.5	1.0	1.0	1.0	1.0	1.0	1.0	1.0	1.0	1.0
27	Howcroft 2017	1.0	1.0	1.0	1.0	0.0	1.0	1.0	1.0	0.5	1.0	1.0	1.0	1.0	1.0	1.0	1.0	1.0	1.0	0.5	1.0	1.0	1.0	1.0	0.0	1.0	0.0	1.0	0.0	1.0	1.0
28	Cheng 2016	0.5	0.0	0.5	0.5	0.5	0.5	0.5	0.5	0.5	0.0	0.5	0.5	0.0	0.5	1.0	0.5	1.0	0.0	0.5	0.0	0.5	0.5	1.0	0.0	1.0	0.5	1.0	0.5	0.5	0.0

R1: Rater 1; R2: Rater 2
